# Patient Perceptions of Natural Orifice Translumenal Surgery

**DOI:** 10.1155/2012/317249

**Published:** 2012-03-05

**Authors:** Melanie E. Tsang, Kirstin Theman, Dale Mercer, Wilma M. Hopman, Lawrence Hookey

**Affiliations:** ^1^Gastrointestinal Diseases Research Unit, Queen's University, Kingston, ON, Canada K7L 2V7; ^2^Department of General Surgery, Queen's University, Kingston, ON, Canada K7L 2V7; ^3^Clinical Research Centre, Kingston General Hospital, Kingston, ON, Canada K7L 2V7; ^4^Department of Community Health and Epidemiology, Queen's University, Kingston, ON, Canada K7L 2V7; ^5^Division of Gastroenterology, Hotel Dieu Hospital, Queen's University, Sydenham 4, Kingston, ON, Canada K7L 5G2

## Abstract

Natural orifice translumenal endoscopic surgery (NOTES) is on the forefront of surgical technique, but existing research has produced mixed results regarding factors associated with interest in the procedure. Our objective was to ascertain patient opinions at a Canadian centre regarding scarless surgery. A survey comprising demographic data (gender, age, body mass index [BMI]), interest in NOTES, impact of increased risk, as well as importance of further research and shorter recovery time was administered to volunteer patients at outpatient general surgery clinics. Nonparametric tests were utilized to examine difference in response by age, sex, BMI, and preexisting scars. Of the 335 participants (57% female, mean age of 54.5 ± 15.9 years, mean BMI of 28.7 ± 6.9), the majority (83%) showed some interest, but this dropped to 38% when additional risk was factored in. Generally, women, those under 50 years of age and those of healthy weight, were more interested than male, older, and/or heavier patients. Most felt that research into NOTES and reduced length of inpatient stay were important (80% and 95%, respectively). Further investigation into objective NOTES outcomes are needed to provide patients adequate data to make an informed choice regarding surgical route.

## 1. Introduction

Natural orifice translumenal endoscopic surgery (NOTES) is on the forefront of surgical technique and is pushing the perceptions and boundaries of abdominal surgery, as laparoscopy did when first introduced. Research continues to progress in this field in both animal and human trials. However, in spite of enthusiasm on behalf of researchers for the technical aspects of NOTES, what will truly lead to its wider implementation will be improved patient outcomes and acceptance. While better patient outcomes (less postoperative pain, fewer—if any—scars, and decreased length of hospital stay) are touted to be the main goal of this technique, it will be some time before hard data are available to assess these. However, patient acceptance of the procedure and its risks can be assessed through surveys in advance of outcomes data.

Though multiple studies have addressed attitudes towards this developing technique, the ability to interpret these variable study results is challenging. Firstly, there is heterogeneity in the questions asked and survey techniques. Secondly, the larger scale studies have come mainly from Europe, thus making direct inferences to a North American population potentially incorrect. Finally, these surveys have emphasized gender and age as variables in assessing interest in NOTES but have not assessed whether previous surgery affects patients perceptions of scars and postsurgical pain. Obesity, surprisingly, has also not been examined previously. It is known that obese patients are at higher risk for developing postoperative hernias and wound infections [1–4] and thus may be a group that could derive significant benefit from NOTES. In this paper, we surveyed a large number of patients at a Canadian centre to assess opinions regarding scarless surgical procedures and whether increased risks would affect their choices. A large sample also allowed for subgroup analyses based on gender, age, and body mass index. 

## 2. Methods

The survey instrument was developed by a team of general surgeons, gastroenterologists, and a statistician. Approval for the study was obtained from the Queen's University Health Sciences & Affiliated Teaching Hospitals Research Ethics Board. A pilot study was performed with 10 people and feedback incorporated into the survey tool. The final survey was comprised of demographic data (age, gender, self-reported height and weight), as well as questions regarding previous surgery and presence and location of scars. Patients were then asked about the importance of scars, bother from scars, interest in scarless surgery, interest in scarless surgery if there were increased complications, acceptable complication rate (from 0% to ≥20%), importance of research into the field, and importance of shorter recovery from surgery. These were all graded on a five-point scale (see the appendix).

All patients attending general surgery outpatient clinics (excluding breast clinics) at Hotel Dieu Hospital—an ambulatory based hospital providing secondary and tertiary care to residents of Kingston, Ontario, and the surrounding area—were invited to fill out a short questionnaire regarding NOTES over a 6-month period in 2008-2009. Surveys were distributed and collected by study hospital staff and deposited in a collection box, which was emptied on a weekly basis to avoid any chance of patient identification. The actual response rate could not be calculated, as the surveys were anonymous and clinic staff did not track the number of patients who were uninterested in responding. However, anecdotal evidence suggests that the patients were generally happy to complete the short survey while they waited. In the event that several appointments were scheduled, patients were asked to complete the survey only once.

### 2.1. Statistics

Data were entered into an Excel spreadsheet designed for the study and entered into SPSS (version 17.0 for Windows, 2009, Chicago, IL) for statistical analysis. Body mass index (BMI) was calculated according to the standard formula of weight (kg) divided by height (metres) squared. BMI was then classified using the standard cutpoints of 18.5–24.9 (healthy weight), 25–29.9 (overweight), 30–34.9 (Obese I), 35–39.9 (Obese II), and ≥35 (Obese III) [5]. Two who were just below the 18.5 threshold were included with the healthy weight group. The three obese groups were also combined for a 3-level analysis. Age was similarly classified as ≤29, 30–49, and ≥50 years.

Data were initially assessed descriptively (mean, standard deviation and range for continuous and ordinal data, frequency and percent for categorical data) and graphed to assess the underlying distribution. Responses to the 5-level Likert scales (1 = no importance, bother, or interest and 5 = extremely important, bothered, or interested) were quantified so that means and standard deviations could be generated. Although the data are ordinal in nature and the use of inferential statistics is not optimal in this situation, they were used for several reasons. First, this was considered preferable to a large volume of chi-square tests. A comparison of medians was also considered but while groups often had similar median values, subtle differences emerged when means were used. Finally, the sample size for the majority of the comparisons was sufficiently substantial to allow the use of inferential statistics in this situation [6]. However, the more conservative nonparametric tests were used to assess all associations.

The associations of age and body mass index with the seven questions were assessed by means of the nonparametric Spearman's correlation. The association of gender and presence of a previous surgical scar (abdominal or nonabdominal) with the seven questions was assessed by means of the Mann-Whitney *U* test, while the association for the three levels of age and BMI were assessed by means of the Kruskal-Wallis test. In order to provide an adequate sample to allow for subgroup analysis, enrolment was aimed at approximately 300 patients. For all analyses, the significance level was set at *P* < 0.05 (two-sided), although results that fell short of statistical significance were noted if they were deemed to be of clinical interest. 

## 3. Results

Three hundred thirty-five patients completed the survey. Demographic and physical characteristics are summarized in Table 1. Nine percent were ≤29 years of age, 26% were 30–49 years, and 64% were ≥50 years; for BMI, 29.9% were at a healthy weight, 34.9% were overweight, and 29.6% were obese (6% were missing height and/or weight). As this was a voluntary, anonymous survey, there were very few missing data (see Table 2). For the few items that were missing, analyses were completed on the subset without missing data, as the type of detailed information typically required for imputation was not collected.

### 3.1. Attitudes towards Scars

**Figure 1:**
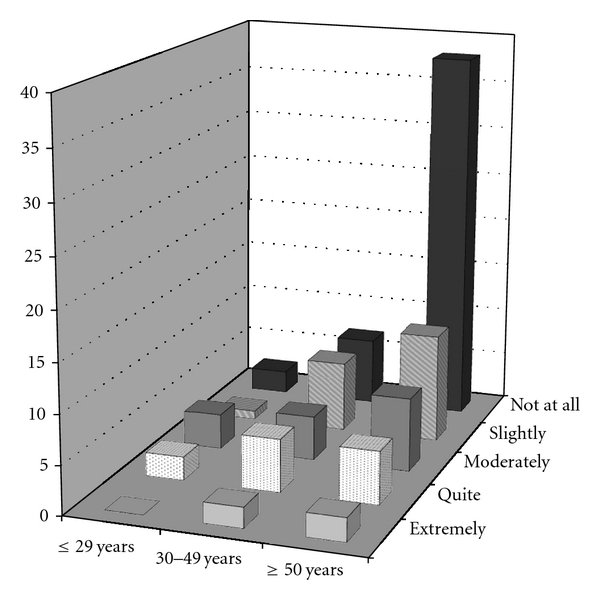
Importance of scars by age category. Percentages are within total sample.

**Figure 2:**
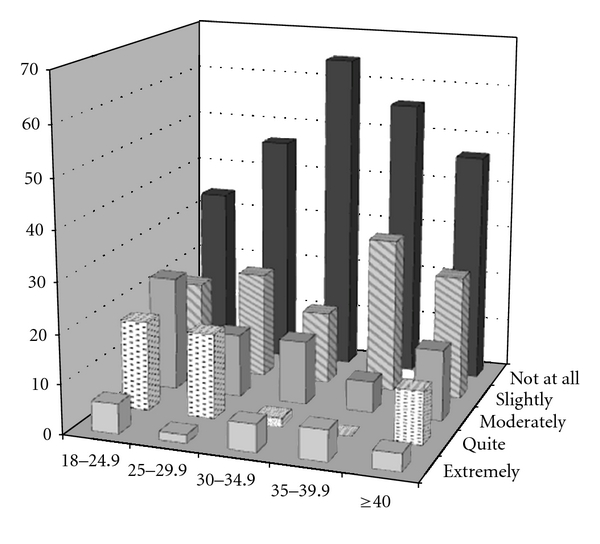
Importance of scars by body mass index category. Percentages are within weight category.

Younger respondents (<50 years of age), females, and those of a healthy weight indicated that cosmetic issues such as scars were more important, as compared to older, male, and heavier respondents (*P* ≤ 0.001 for all three comparisons) (Table 3). Amongst all surveyed, 87% of respondents had some type of scar. Of these, 58% indicated that it did not bother them at all, but 9.9% indicated that they were bothered quite a bit or extremely by their scar(s). Women placed significantly greater importance on abdominal scars than men and were more greatly impacted by them; fifty-six percent of women were bothered by some degree by their current scars as compared with 23% of men (*P* < 0.001). Age (as a continuous variable) was negatively correlated with the importance and impact of abdominal scars; in other words, as age increased, the importance and impact of abdominal scars decreased (*P* < 0.001, see Figure 1 for importance). Similarly, as BMI increased, the importance of abdominal scars significantly decreased (*P* < 0.001, Figure 2.) 

### 3.2. Interest in Scarless Surgery and Acceptance of Complication Rates

The majority (83%) had at least some interest in a surgery that would leave no scars. The two younger groups were more interested than those over 50 years (*P* = 0.001), with those between 30 and 49 years remaining the most interested in the face of increased risk (*P* = 0.036). The two younger groups were comfortable with a risk up to 10%, while the older group was more conservative and was more comfortable with a risk close to 5% (*P* = 0.003). There were also gender differences in the level of interest, with women expressing more interest than men (*P* = 0.021). This difference disappeared when the question of risk was added (*P* = 0.192), although the women tended to accept an increased risk of close to 10%, while the men were closer to 5% (*P* = 0.059).

Level of interest in NOTES was not significantly related to BMI, nor was acceptance of increased rate of complication, or the amount of acceptable risk. However, for all three questions, those at a healthy weight had the highest scores, suggesting more interest and less concern about risk. Those without previous abdominal scars were more interested in NOTES than those with scars (*P* = 0.049), but both groups lost interest when presented with increased risk. The presence of other scars had little association with the responses to the three questions. 

### 3.3. Research into NOTES

Over 80% of respondents felt that research into scarless surgery was of some importance, with 30.4% rating it as quite or extremely important. With age as a continuous variable, the Spearman correlation suggested a negative but significant association (rho = −.205, *P* < 0.001); using the categorical variable, those in the age group of 30–49 years rated research as more important than the younger or older groups (*P* = 0.040). BMI was also negatively and significantly associated with importance when using the continuous variable (rho = −.149, *P* = 0.009), but fell just short of significance when using the categorical variable (*P* = 0.066), although it was the healthy weight group that was more likely to rate it as important. Women rated it as more important than men, although it fell short of significance (*P* = 0.084). Presence of abdominal or other scars had little association with the ratings of importance.

### 3.4. Shorter Hospital Stay

One of the key proposed benefits of NOTES is a decreased length of stay in the hospital. Very few (only 5.1%) indicated that a shorter hospital stay was not important, with 64.8% indicating that it was quite or extremely important. There was a weak, negative association with age using the Spearman correlation (rho = −.109, *P* = 0.049), but this was no longer significant when using the categorical data (*P* = 0.537). Sex, BMI, and presence of scars also had little association with the importance of shorter in-hospital recovery time.

## 4. Discussion

Here, we captured the opinions of 335 North American patients to obtain their views on this developing technique. Several patient surveys have attempted to characterize those who would be most interested in this new method. Studies published to date have variable results, perhaps related to the population surveyed and questions asked. Some surveys have shown that patients prefer NOTES to laparoscopic surgery due to its improved cosmetic result with the potential for decreased pain also holding appeal in some studies [7–10]. However, patients consistently had decreased interest as the potential rate of complication increased [7, 9]. Single port surgery (SPS) is a minimally invasive form of laparoscopic surgery and a large-scale British study (*n* = 750) comparing patient views on it and NOTES showed that SPS was significantly preferred over open surgery and NOTES [11]. Although experts often point to women as being a target group who would be interested in NOTES [12], studies looking at the effect of gender on opinions of NOTES have led to conflicting results. Varadarajulu et al. did not find a significant preference by women for NOTES compared to men [9]. Further to this, surveys targeted at women in the context of transvaginal NOTES have had variable results. Sixty-eight percent of women were interested in NOTES in a study by Peterson et al. [8]. However, in an Australian study, three quarters of surveyed women were neutral or unhappy about transvaginal NOTES compared with standard laparoscopic surgery [13].

In keeping with the results of previous surveys, women were significantly more concerned with the cosmetic results of surgery and were more bothered by current scars. NOTES, being a “scarless” method, would allay this concern. In addition, female patients are anatomically more versatile candidates for NOTES, with the potential for a transvaginal approach. Our study did support the theory that women would be more interested in NOTES than men, but this association was lost when additional risk was factored into the equation. Those under 50 years of age rated a scarless method as being more important and expressed more interest, even in the face of increased risk.

Although there was a high interest in the concept of NOTES (83% showed at least slight interest), this dropped to 38% when an increased complication risk was proposed compared to traditional techniques. However, this remains a significant proportion of the surveyed population, and provides impetus to further research and development in this field to make it a safe alternative to laparoscopic and open surgery. This is borne out in our data where 81% of patients felt that research into NOTES held some level of importance.

One of the groups in the position to benefit the most from NOTES is obese patients, though our data show that level of interest in the technique is significantly and *negatively* associated with BMI, such that those of healthy weight expressed greater interest. Obese patients are especially at risk for hernias after transabdominal surgery [4–6] and NOTES could mitigate this risk. The lack of abdominal wall incisions could also lead to earlier postoperative mobilization, better lung ventilation, decreased wound infections, all of which would lead to decreased length of hospital stay [12]. Furthermore, NOTES-assisted bariatric surgery has now been successfully attempted [14] and in the authors' opinion is one of the prime areas for NOTES development. Hence, further objective data and education will be necessary to garner the interest and support of this population in this new technique.

Though the capital investment required for the development and adoption of any new technique is significant, the potential for cost savings in projected shorter hospital stays could offset the cost. Ninety-five percent of patients indicated that a shorter in-hospital stay was important to them, adding to the attractiveness of this aspect of NOTES. The reasons behind patient interest in shorter length of hospital stay were not explored further but could include less time away from home and increased awareness of hospital acquired infections. Third party payers (insurance companies and governments) would certainly also be interested in a technique that reduces hospital stay. In addition, it has been proposed that once further developed NOTES would not require a traditional operating room, thus altering hospital utilization further [15].

The current study has some limitations. By dint of the survey population being from surgical clinics, a large proportion already had scars, which may have skewed the results. While the self-administered survey prevented any bias that might have stemmed from a personal interview, patients were unable to ask for any more detail regarding the technique and complications than was included in the survey. For example, when presented with potential complications such as dyspareunia and infertility, women may in fact be less interested in the transvaginal approach of NOTES. Qualitative data collection may provide more insight into the subtleties of patient concerns. This could also be extended to multiple centres to capture regional differences in opinion as the present study was performed in a single centre.

Our results show that there is significant Canadian patient interest in NOTES. The technique is still in its early stage of acceptance, but our data lend support to this endeavour. Clearly, once techniques are further refined, hard data including complication rates, length of stay, and post-operative pain will be necessary to assess its utility and give patients adequate information for an informed choice.

## Figures and Tables

**Table 1:** Patient demographics.

Characteristic	Mean (standard deviation) [range]
Age	54.5 (15.9) [17–88]
Height (cm)	168.5 (10.4) [127–198]
Weight (kg)	82.1 (22.8) [38.1–199.6]
BMI	28.7 (6.9) [17.9–64.3]

	Frequency (Percent)

Male	144 (43.0)
Existing abdominal scar	209 (62.4)
Other major nonabdominal scar	158 (47.2)

**Table 2:** Missing data (*n* = 335).

Variable	Missing
	*N* (%)
Age	1 (0.3)
Gender	1 (0.3)
BMI	20 (6.0)
Previous abdominal scar	3 (0.9)
Major non-abdominal scars	11 (3.3)
Importance of scars	2 (0.6)
Impact of current scars	1 (0.3)
Interest in no scar surgery	2 (0.6)
Interest if increased complications	8 (2.4)
Reasonable risk	18 (5.4)
Importance of research	12 (3.6)
Importance of shorter stay	9 (2.7)

**Table 3:** Associations between patient characteristics and opinions. Please see the appendix for detailed responses. Scales are scored from 1–5, with 1 representing no importance, bother, interest, or no increased acceptable risk; 5 = extremely important, bothered, interested, and a 20% increased risk. Values represent means and standard deviations, but *P* values are based on the Mann-Whitney U or the Kruskal-Wallis as appropriate.

Characteristic	Importance	Feel about current scars*	Interest in surgery with no scars	Even if increased risk of infection	How much additional risk	Importance of research	Importance of shorter recovery time
Age in years							
≤29	2.7 (1.1)	1.9 (1.1)	3.3 (1.4)	1.6 (0.9)	1.9 (0.9)	2.9 (1.3)	3.9 (1.1)
30–49	2.6 (1.3)	2.1 (1.2)	3.3 (1.2)	1.9 (1.1)	2.0 (1.1)	3.1 (1.2)	3.9 (1.1)
50+	1.8 (1.2)	1.6 (1.0)	2.8 (1.3)	1.6 (1.0)	1.6 (1.0)	2.7 (1.3)	3.7 (1.2)
*P* value	<0.001	<0.001	0.001	0.036	0.003	0.040	0.537

Sex							
Female	2.4 (1.3)	2.1 (1.2)	3.1 (1.3)	1.8 (1.1)	1.9 (1.1)	2.9 (1.3)	3.8 (1.1)
Male	1.7 (1.1)	1.4 (0.8)	2.8 (1.3)	1.6 (0.9)	1.6 (0.8)	2.7 (1.2)	3.7 (1.2)
*P* value	<0.001	<0.001	0.021	0.192	0.059	0.084	0.363

BMI Category							
Healthy	2.4 (1.3)	1.8 (1.0)	3.1 (1.3)	1.8 (1.1)	2.0 (1.2)	3.0 (1.2)	3.8 (1.1)
Overweight	2.1 (1.2)	1.9 (1.2)	2.9 (1.3)	1.6 (0.9)	1.7 (0.9)	2.8 (1.3)	3.7 (1.0)
Obese	1.8 (1.1)	1.6 (1.0)	2.9 (1.2)	1.6 (1.0)	1.6 (0.9)	2.6 (1.3)	3.7 (1.3)
*P* value	0.001	0.123	0.297	0.272	0.253	0.066	0.786

Abdominal Scar							
No	2.3 (1.3)	—	3.1 (1.3)	1.7 (0.9)	1.8 (0.9)	3.0 (1.2)	3.9 (1.1)
Yes	2.0 (1.2)	1.8 (1.1)	2.8 (1.3)	1.7 (1.1)	1.7 (1.1)	2.8 (1.3)	3.7 (1.1)
*P* value	0.071	—	0.049	0.431	0.203	0.222	0.104

Other Scar							
No	2.0 (1.2)	—	2.9 (1.3)	1.7 (1.0)	1.7 (0.9)	2.8 (1.3)	3.8 (1.1)
Yes	2.1 (1.3)	1.9 (1.2)	3.0 (1.3)	1.7 (1.1)	1.8 (1.1)	2.9 (1.3)	3.8 (1.2)
*P* value	0.527	—	0.416	0.964	0.939	0.275	0.740

*Responses are based on the subset with scars.

## Appendix

### Survey Instrument

Survey of Opinions Regarding a New Surgical Technique

Age: ……years
Sex: 
  Male (  )
  Female (  )
Height:  … (in feet and inches) or …(in centimetres)
Weight: … … (in pounds) or …… (in kilograms)
Do you have an abdominal scar from a previous surgery?
  Yes (  )
  No (  )
Do you have any other major scars?
  Yes (  )
  No (  )
 If yes, where? … …………………………

For the following questions, please place a check mark in the box that  corresponds best with what you think

1. How important are cosmetic issues, like scars, to you in abdominal surgery?
  Not at all important (  )
  Slightly important (  )
  Moderately important (  )
  Quite important (  )
  Extremely important (  )
2. How do you feel about the scars you have?
  Not applicable, no scars (  )
  Do not bother me at all (  )
  Bother me slightly (  )
  Bother me moderately (  )
  Bother me quite a bit (  )
  Extremely bothered (  )
3. Would you be interested in a surgery that would leave no scars?
  Not interested (  )
  Slightly interested (  )
  Moderately interested (  )
  Quite interested (  )
  Extremely interested (  )
4. Would you be interested in a surgery that would leave no scars even if there was an increased risk of complications such as infection inside your abdomen?
  Not interested (  )
  Slightly interested (  )
  Moderately interested (  )
  Quite interested (  )
  Extremely interested (  )
5. How much increased risk would you be comfortable with if the surgery would leave no scar? For example, if you pick 5%, you are indicating that you'd be comfortable with a 5 in 100 chance of having a complication such as infection just to have a scarless surgery.
  None, would not have scarless surgery (  )
  5% (  )
  10% (  )
  15% (  )
  20% or more (  )
6. How would you rate the importance of further research and investment into scarless surgery?
  Not important at all (  )
  Slightly important (  )
  Moderately important (  )
  Quite important (  )
  Extremely important (  )
7. How important is a shorter recovery time (time spent in hospital recuperating from surgery) to you?
  Not important at all (  )
  Slightly important (  )
  Moderately important (  )
  Quite important (  )
  Extremely important (  )
